# Tumor Response Evaluation Using iRECIST: Feasibility and Reliability of Manual Versus Software-Assisted Assessments

**DOI:** 10.3390/cancers16050993

**Published:** 2024-02-29

**Authors:** Inka Ristow, Lennart Well, Nis Jesper Wiese, Malte Warncke, Joseph Tintelnot, Amir Karimzadeh, Daniel Koehler, Gerhard Adam, Peter Bannas, Markus Sauer

**Affiliations:** 1 Department of Diagnostic and Interventional Radiology and Nuclear Medicine, University Medical Center Hamburg-Eppendorf, 20246 Hamburg, Germanym.sauer@uke.de (M.S.); 2 Department of Oncology and Hematology, Bone Marrow Transplantation with Section Pneumology, University Medical Center Hamburg-Eppendorf, 20246 Hamburg, Germany

**Keywords:** computed tomography, imaging, iRECIST, RECIST, manual, response assessment, oncology, reading time, software, treatment response

## Abstract

**Simple Summary:**

Quantitative assessment of the therapy response in oncological patients undergoing chemo- or immunotherapy is becoming increasingly important not only in the context of clinical studies but also in clinical routine. To facilitate the sometimes complex and time-consuming oncological response assessment, dedicated software solutions, e.g., according to (i)RECIST, have been developed. Considering the higher degree of complexity of iRECIST, we investigated the benefits of software-assisted assessments compared to manual approaches with respect to reader agreement, error rate, and reading time. iRECIST assessments were more feasible and reliable when supported by dedicated software. We conclude that oncologic response assessment in clinical trials should be performed software-assisted rather than manually.

**Abstract:**

Objectives: To compare the feasibility and reliability of manual versus software-assisted assessments of computed tomography scans according to iRECIST in patients undergoing immune-based cancer treatment. Methods: Computed tomography scans of 30 tumor patients undergoing cancer treatment were evaluated by four independent radiologists at baseline (BL) and two follow-ups (FU), resulting in a total of 360 tumor assessments (120 each at BL/FU1/FU2). After image interpretation, tumor burden and response status were either calculated manually or semi-automatically as defined by software, respectively. The reading time, calculated sum of longest diameter (SLD), and tumor response (e.g., “iStable Disease”) were determined for each assessment. After complete data collection, a consensus reading among the four readers was performed to establish a reference standard for the correct response assignments. The reading times, error rates, and inter-reader agreement on SLDs were statistically compared between the manual versus software-assisted approaches. Results: The reading time was significantly longer for the manual versus software-assisted assessments at both follow-ups (median [interquartile range] FU1: 4.00 min [2.17 min] vs. 2.50 min [1.00 min]; FU2: 3.75 min [1.88 min] vs. 2.00 min [1.50 min]; both *p* < 0.001). Regarding reliability, 2.5% of all the response assessments were incorrect at FU1 (3.3% manual; 0% software-assisted), which increased to 5.8% at FU2 (10% manual; 1.7% software-assisted), demonstrating higher error rates for manual readings. Quantitative SLD inter-reader agreement was inferior for the manual compared to the software-assisted assessments at both FUs (FU1: ICC = 0.91 vs. 0.93; FU2: ICC = 0.75 vs. 0.86). Conclusions: Software-assisted assessments may facilitate the iRECIST response evaluation of cancer patients in clinical routine by decreasing the reading time and reducing response misclassifications.

## 1. Introduction

The response evaluation criteria in solid tumors (RECIST) were proposed more than 20 years ago to establish objective guidelines to assess cancer treatment response based on cross-sectional imaging, especially computed tomography (CT) [1]. Since then, numerous revisions and adaptations have been published, of which RECIST version 1.1 is still the most commonly used classification in clinical trials [2,3]. For certain tumor entities, specific modifications were introduced (e.g., modified RECIST for hepatocellular carcinoma or mesothelioma) [4,5,6], and the emergence of new therapeutic targets in cancer treatment has led to further adaptations in response evaluation.

The adapted RECIST 1.1 for immune-based therapeutics (iRECIST) is becoming more important given the increasing number of clinical trials that are conducted to evaluate the treatment response of solid tumors under immunotherapy [7]. iRECIST offers a more comprehensive approach for standardized tumor response assessments taking into account the broad spectrum of possible responses to immunotherapy [8]. iRECIST follows the RECIST 1.1 recommendations at the lesion level, i.e., measurement technique, size criteria, disease selection, and categorization. However, compared to RECIST 1.1, which allows for the determination of four possible response states [progressive disease (PD), stable disease (SD), partial and complete remission (PR/CR)], iRECIST additionally introduced “unconfirmed” and “confirmed progressive disease” (iUPD/iCPD). Hereby, potential pseudo-progressions, which frequently occur during immunotherapy, shall be addressed. Pathophysiologically, pseudo-progression is attributable to intra- and peritumoral infiltration of immune cells (such as CD4 T cells, CD8 T cells, and macrophages), which promote the occurrence of tumor necrosis, hemorrhage, and edema [9,10]. However, pseudo-progressions may also occur during conventional non-immunotherapies, and therefore the extended application of iRECIST should be a matter of discussion.

Determining the correct disease state of follow-ups according to iRECIST can be challenging and requires profound knowledge of the interpretation rules [11]. Among different clinical trial sites, there are numerous technical approaches on how to perform tumor response assessments, and the current guidelines offer no instruction regarding the technical implementation of RECIST. A relevant number of evaluations is still based on manually generated tables where measurements from previous radiological reports are documented and evaluated. This approach bears several error sources, such as the neglect of previous examinations, misassignments, or miscalculations. 

Various software tools with different degrees of automation have been introduced to minimize misinterpretations [12,13]. Concepts of fully automated approaches including image interpretation and segmentation have been proposed [14,15] but have not been implemented in clinical trials so far. In our study, image interpretation and lesion definition were performed solely by a radiologist, while the assessment software provided automatic tumor burden calculation and the determination of the response according to iRECIST, hereby representing a semi-automated, software-assisted approach. 

The aim of this study was to compare the feasibility, reliability, and agreement between manual and software-assisted response assessments using the complex iRECIST criteria.

## 2. Material and Methods

This retrospective study at a single tertiary center was approved by the local Institutional Review Board (Ärztekammer Hamburg, Germany; approval date: 28 July 2023) with a waiver of informed consent (2023-300358-WF). All procedures were in accordance with the principles of the 1964 Declaration of Helsinki and its later amendments. All patient data and CT images were anonymized before tumor response evaluation. Previously conducted iRECIST assessments for the actual clinical trials were disregarded for this study.

### 2.1. Study Population

To ensure eligibility for iRECIST assessments, we randomly selected 30 patients (12 male; 18 female; mean age 58.9 ± 11.3 years) from our database who had previously participated in a clinical trial that investigated immune-based therapeutics at our local cancer center. Patients were included if at least two follow-ups after baseline imaging were available, resulting in 90 CT scans to be assessed. The tumor entities were as follows: 7× colorectal cancer, 7× breast cancer, 5× ovarian cancer, 4× testicular cancer, 2× cholangiocellular carcinoma, 2× lung cancer, 2× esophageal cancer, 1× hemangiopericytoma. Both first-line as well as subsequent-line immunotherapies were considered.

### 2.2. Imaging

In-house CT scans were performed in all included patients according to the respective protocol specifications of the originally performed study. All included examinations were contrast-enhanced scans in the portal venous phase of the thorax, abdomen, and pelvis with reconstruction in 5 mm and 1 mm slice thicknesses and multiplanar reformations in sagittal and coronal orientation. Scan delay time from the injection of the contrast medium was 90 s.

### 2.3. Data Analysis

**Figure 1:**
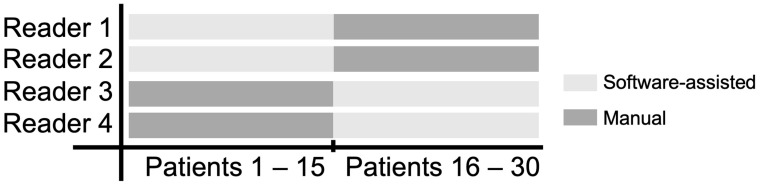
CT data analysis scheme to assess tumor response according to iRECIST. In order to minimize the impact of the individual reader, each of the four readers performed half of their readings manually (dark grey) and software-assisted (light grey), respectively.

Four radiologists (L.W., N.J.W., M.W., M.S.) with 5–10 years of expertise in oncologic imaging who routinely perform iRECIST tumor assessments at the institution participated in this study. Before the evaluation, each reader was handed the current iRECIST guidelines [8], and the key aspects were repeated to ensure an equal level of knowledge for the evaluation. To minimize the influence of the individual reader’s performance, each reader first assessed half of the patients manually and the second half software-assisted. The evaluation scheme is visualized in Figure 1. Each reader was provided with a list indicating which examinations were to be assessed in the respective manner. Computed tomography scans were first reviewed by the respective reader using the institutional picture archiving and communications system (PACS, Centricity Universal Viewer, GE Healthcare™, Chicago, IL, USA). Baseline (BL), follow-up 1 (FU1), and follow-up 2 (FU2) assessments were performed in blocks with at least four weeks in between, thus requiring the readers to refocus at each follow-up time point.

### 2.4. Reading Time Assessment

Prior to the actual iRECIST assessments, the readers were given unlimited time to screen each CT scan. Each iRECIST reading time measurement was started by the respective reader when either the manual template or the assessment software was opened, and it was stopped when the manual template or the software-based results report was exported.

#### 2.4.1. Manual iRECIST Assessment

For the manual iRECIST assessment, a digital template based on an Excel spreadsheet (Version 16.80, Microsoft Corporation, Seattle, WA, USA) was provided on a separate computer screen (Supplementary Material Figure S1), which was completed in parallel with the image data opened via the PACS viewer used in clinical routine.

Target and non-target lesions with respective measurements were defined by the readers according to iRECIST [8]. During follow-up, the lesions that had been previously determined and documented in the template had to be re-evaluated, and the overall response, including the calculation of the sum of longest diameters (SLD), was determined manually.

#### 2.4.2. Software-Assisted iRECIST Assessment

For the software-assisted assessment, Mint Lesion^TM^ (V. 3.7; Mint Medical GmbH, Heidelberg, Germany) was used. For the baseline study, target and non-target lesions first had to be annotated in the software and measured using the tools provided. The software offers guidance on how to measure according to the iRECIST guidelines and points out deviations and errors (e.g., one-dimensional measurement of lymph nodes or an excess of lesions). However, since the measurements must be performed manually, we followed Folio et al. and therefore use the term semi-automatic approach [16]. 

During follow-up examinations, the previously defined findings were re-evaluated using the tools provided by the software. The overall response was automatically assessed based on the measurements, and the results report, including percentage changes, was provided as a standardized and exportable PDF table.

### 2.5. Analysis of Response Assessments

Each reader determined and documented the overall iRECIST response for the two follow-ups (2 × 120 assessments) using either the manual (2 × 60 assessments) or software-based approach (2 × 60 assessments).

After all readings were completed, all response evaluations were reviewed together by all readers as a consensus reading to determine the correct answer, which was set as the reference standard. In the case of multiple correct options, e.g., due to a different choice of target lesions, legitimate deviations were accepted. For the final analysis, illegitimate deviations from the reference standard were counted for both follow-ups.

### 2.6. Quantitative Inter-Reader Agreement

At both follow-ups, each reader calculated the percentage change in the SLD for each assessment, which is one parameter of the overall response. The extent of change provides information about the magnitude of the development of the tumor burden. Agreement regarding the SLD response was analyzed for all assessments and the respective groups (manual vs. software-assisted).

### 2.7. Statistical Analysis

Normal distribution of continuous data was evaluated using the Kolmogorov–Smirnov test. Normally distributed continuous data are presented as mean ± standard deviation. Non-normally distributed continuous data are given as median with interquartile range (IQR). 

Friedmann’s test was conducted to examine differences in the reading time for the manual versus software-assisted assessments. The Mann–Whitney U test was applied for post hoc testing, i.e., pairwise comparisons of reading time between manual versus software-assisted assessments at BL, FU1, and FU2.

Regarding incorrect response assessments, the degree of agreement is reported descriptively for both follow-ups. Regarding the quantitative inter-reader agreement, Bland-Altman analyses were conducted between the manual and software-assisted assessments followed by a calculation of intraclass correlation coefficients to assess the interobserver correlation of the software-assisted vs. manual assessments.

All tests were two-sided. *p*-values < 0.05 were considered statistically significant. Statistical analysis was performed using GraphPad Prism 5.0 for Windows (GraphPad Software Inc., La Jolla, CA, USA) and SPSS Version 27.0 (IBM Corp. IBM SPSS Statistics for Windows, Armonk, NY, USA).

## 3. Results

### 3.1. Reading Time Assessment

**Figure 2:**
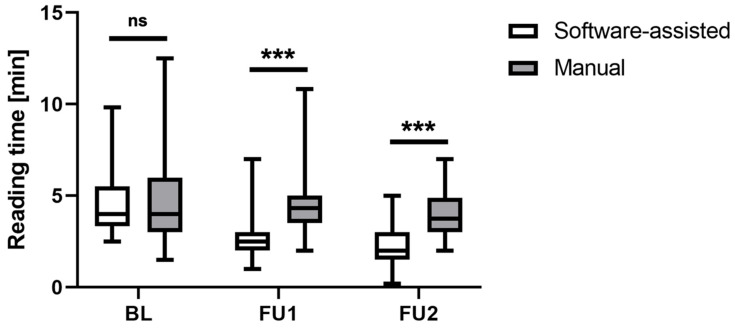
Comparison of reading times for the iRECIST assessment at different study time points revealed significantly shorter reading times for the software-assisted approach compared to the manual approach at both follow-ups. Box plots show median and quartile ranges of the reading times in seconds. BL = baseline, FU1 = follow-up 1, FU2 = follow-up 2, ns = non-significant, *** = *p* < 0.001.

The reading times at BL, FU1, and FU2 are visualized in Figure 2.

Regarding the baseline assessment, the median reading time (with 25% and 75% quantiles) was 4 min (3.33; 5.50) for the software-assisted assessment and 4 min (3.00; 5.98) for the manual assessment. 

For FU1, the median reading time was 2.5 min (2.00; 3.00) for the software-assisted assessment and 4.30 s (3.50; 5.00) for the manual assessment, and for FU2, it was 2 min (1.5; 3) and 3.8 min (3.0; 4.9), respectively. Friedmann’s test revealed significant differences in the reading times for the manual vs. software-assisted assessments (χ^2^ = 127.42; N = 60; df = 5; *p* < 0.001). While the post hoc testing showed no significant group difference at BL (*p* = 0.935), the reading times at FU1 and FU2 were significantly shorter for the software-assisted approach compared to the manual assessment (both *p* < 0.001).

### 3.2. Analysis of Response Assessments

The number of incorrect response assessments according to the reference standard is given in Table 1. The error rate in FU1 was 3.3% for the manual assessment, while no errors were observed for the software-assisted approach. This trend deteriorated in FU2, where the error rate for the manual assessment increased to 10%, while the error rate for the software-assisted approach was only at 1.7%.

**Figure 3:**
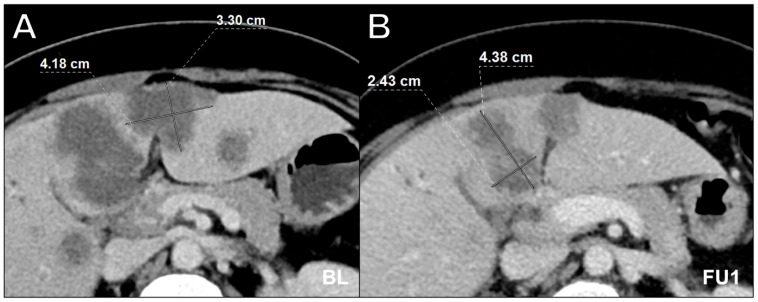
Axial contrast-enhanced computed tomography scan in portal venous phase in a 64-year-old male with multiple hepatic metastases in colorectal carcinoma at baseline (**A**) and follow-up 1 (**B**) showing incorrect lesion assessment at follow-up 1. Appropriate documentation is essential, especially when there are multiple target lesion candidates. While the software-assisted approach offers side-by-side assessment with structured lesion documentation, reader 3, who performed the manual assessment, mixed up lesions at follow-up 1, resulting in an incorrect overall response compared to the consensus reading. It is likely that the presentation state was not correctly stored in the PACS when the baseline measurement of the lesions was performed. BL = baseline, FU1 = follow-up 1.

**Figure 4:**
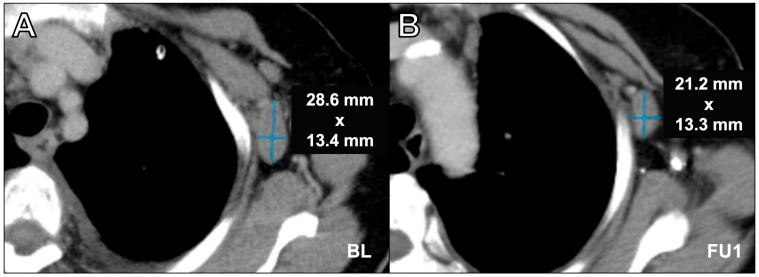
Axial contrast-enhanced computed tomography scan in portal venous phase in a 76-year-old female with breast cancer and axillary lymph node metastasis assessment at baseline (**A**) and at follow-up 1 (**B**). Incorrect lymph node assessment was performed at follow-up 1 by using the long axis. For adequate assessment, it is crucial to measure the lymph nodes in two dimensions, as only the short axis is the relevant value. While the assessment software demands two-dimensional measurements, manual measurement deviating from the short axis is a common source of error. Furthermore, neglecting the longest diameter when it was located on a different plane of the y-axis was an understandable source of error in our study (see the position of the aortic arch in the given example). BL = baseline, FU1 = follow-up 1.

The retrospective analysis of incorrect assessments revealed several sources of error. The manual assessments revealed documentation errors, such as incorrect identification of target lesions (as shown in Figure 3) as well as misapplied iRECIST guidelines. In the software-assisted assessments, only one case of incorrect lesion measurement at the wrong level was detected (Figure 4).

### 3.3. Interobserver Correlation

The interobserver correlation of the quantitative SLD measurements was good to excellent for both the manual and the software-assisted approaches at both time points but decreased from FU1 to FU2. However, the software-assisted SLD measurements correlated more strongly compared to the manually assessed SLD measurements. At FU1, the intraclass correlation coefficient (ICC) of the manual assessment was 0.91 (CI 0.82–0.96), and for the software-assisted assessment, it was 0.93 (CI 0.86–0.97). At FU2, the ICC of the manual assessment was 0.75 (CI 0.42–0.83), and for the software-assisted assessment, it was 0.86 (CI 0.42–0.97). 

### 3.4. Quantitative Inter-Reader Agreement

**Figure 5:**
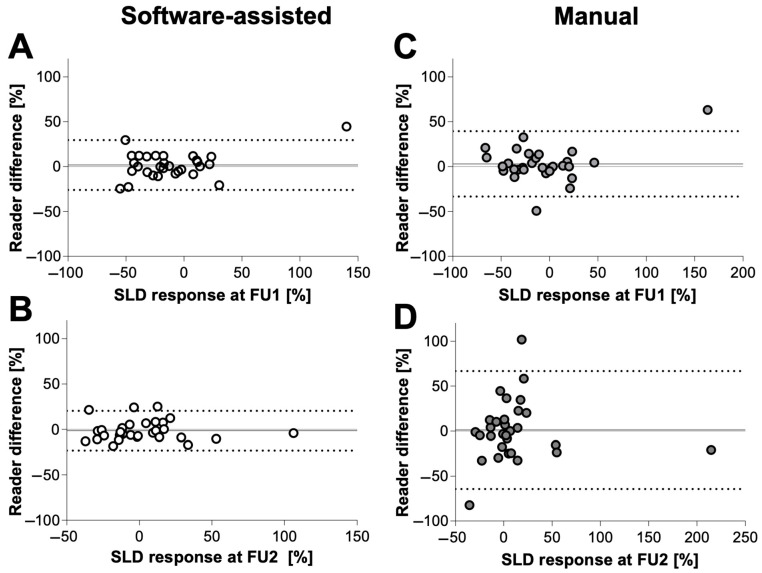
Bland–Altman analyses showing reader difference in % of SLD of software-assisted (**left**) vs. manual (**right**) tumor response according to iRECIST at follow-up 1 (**A**,**C**) and follow-up 2 (**B**,**D**). The software-assisted reading evaluation achieved better agreement among readers. The dotted lines indicate limits of agreement, and the middle solid line indicates the mean bias of diameter measurements. FU1 = follow-up 1, FU2 = follow-up 2, SLD = sum of longest diameters.

Regarding the SLD assessments, there were no significant differences at FU1. At FU2, the mean difference between the two readers was significantly lower for the software-assisted compared with the manual assessments at FU2, while there were no significant differences at FU1. The results of Bland–Altman analyses are given in Table 2 and visualized in Figure 5.

## 4. Discussion

The aim of this study was to investigate the impact of dedicated assessment software on iRECIST evaluations concerning feasibility and reliability. Aside from RECIST 1.1 [13,17], to our knowledge, there is no previous scientific evidence about the impact of software-based methods on iRECIST assessments. Our results reveal that software-assisted iRECIST readings can be performed with (I) less time effort, (II) fewer incorrect response assessments, and (III) a higher inter-reader agreement compared to manual approaches.

To address the problem of significant time efforts caused by RECIST assessments, several workgroups investigated if assessments may even be delegated to radiology technologists [18,19]. Demonstrating that software-assisted readings save a significant amount of time compared to manual procedures in our study, we would contradict and propose that high-quality “one-stop-shop” readings by the radiologist during clinical routine are achievable and delegation is unnecessary. Concerns regarding inadequate assessments due to the convenience of readers (e.g., by selecting only a few target lesions limiting the representativeness of the results [20]) should be addressed in the same manner and emphasize the advantage of quick and easy readings. 

The reliability of cancer therapy response assessments is a long-term ongoing debate but is rarely the subject of scientific investigation [21,22]. With respect to RECIST 1.1, several studies have shown that the correct response assessments depend, amongst others, on the selection of target lesions [23,24], but there is little knowledge about the impact of the assessment technique on the rate of correct assessments. Our data demonstrated an error rate of 10.0% for the manual assessment vs. 1.7% for the software-assisted assessment at the second FU, hereby highlighting the serious impact of the assessment technique on the frequency of false assessments.

It is worth mentioning that, in our study, we chose to allow for legitimate deviations (e.g., caused by different target lesion selection) to be able to focus on undeniably false assessments due to incorrect measurement, miscalculation, or lesion confounding. Divergent assessments may reflect the complexity of different tumor responses; hence, iRECIST offers a broader spectrum of possible responses (e.g., unconfirmed progressive disease), and consequently, in certain treatment courses, there will be no definitive truth. Yet, despite the complexity, divergent assessments due to formal errors are unacceptable in the context of cancer therapy, and our results support the use of a software-assisted assessment technique to avoid unnecessary treatment adjustments.

To further investigate the assessment reliability in terms of inter-reader agreement, we used the SLD calculated according to the iRECIST criteria as a quantitative parameter representing the patient’s tumor burden. We demonstrated good overall agreement for both the manual and software-assisted readers, though it was higher in the latter (ICC 0.91 vs. 0.93, respectively). Since already small SLD deviations can have a decisive impact in this context, we think that this finding underlines the basic functionality of (i)RECIST, supporting its widespread application. However, at FU2, inter-reader agreement decreased for the software-assisted assessments (ICC 0.86) and even more for the manual readings (ICC 0.75), indicating a trend towards increasing discrepancy in long-term therapies. In line with these findings, Karmakar et al. demonstrated a trend toward decreasing reader agreements in follow-up tumor assessments [25] and therefore proposed standardized checklists to reduce methodological errors. Especially concerning iRECIST, the correct assessment requires a sound knowledge of the basic rules as well as a thorough review of previous exams, both of which are significantly simplified by software assistance. Furthermore, the proposed checklist to reduce methodological errors is already implemented in available software solutions. Hereby, inter-reader agreement and overall assessment reliability can be improved.

Our study has the following limitations: The focus of our analysis was on the optimal methodical implementation of iRECIST rather than its actual diagnostic performance for cancer therapy evaluation. Therefore, our restriction to three examination time points per patient seems acceptable. Yet, especially in cases of unconfirmed progressions, additional follow-ups would be desirable, and further analysis of the impact of long-term treatment courses on iRECIST evaluations should be considered. Because of the complexity of our four-reader design, we limited our study population to 30 patients; however, further studies in larger cohorts are desirable. Additionally, further analysis of the impact of first- vs. subsequent-line immunotherapies, which were mixed in this study, should be considered. Moreover, comparing RECIST 1.1 and iRECIST could provide further insights into determining the feasibility and reliability of software-assisted vs. manual response assessments. Regarding the impact of iRECIST and software-assisted assessments on the clinical outcome of patients, further correlations with parameters such as morbidity and survival are needed, which were not considered in this study.

## 5. Conclusions

In conclusion, the results of our study demonstrate the better feasibility and increased reliability of iRECIST assessments when supported by dedicated software compared to manual approaches. To ensure optimal oncological response evaluation in clinical trials, software-assisted solutions for oncological response assessment should be used instead of manual approaches.

## Figures and Tables

**Table 1:** Incorrect iRECIST response assessments according to the reference standard.

Examination Timepoint	Software-Assisted	Manual
FU1	0/60 (0%)	2/60 (3.3%)
FU2	1/60 (1.7%)	6/60 (10%)

**Table 2:** Quantitative inter-reader iRECIST agreement regarding SLD assessments.

		Software-Assisted	Manual	*p*-Value
Mean difference (%) ± SD	FU1	10.2 ± 0.9	11.6 ± 2.1	0.56
	FU2	8.9 ± 6.5	23.4 ± 23.2	0.001
Bias ± SD	FU1	1.8 ± 14.2	3.0 ± 18.6	
	FU2	−1.4 ± 11.1	1.20 ± 33.4	
95% limits of agreement	FU1	−26.1 to 29.6	−33.41 to 39.4	
	FU2	−23.2 to 20.4	−64.3 to 66.7	

## Data Availability

The datasets used and/or analyzed during the current study are available from the corresponding author upon reasonable request.

## Supplementary Materials

The following supporting information can be downloaded at: https://www.mdpi.com/article/10.3390/cancers16050993/s1, Figure S1: Tumor response assessment according to iRECIST criteria.

## Abbreviations

BL = baseline, CI = confidence interval, CR = complete remission, CT = computed tomography, FU1 = follow-up 1, FU2 = follow-up 2, iRECIST = immune response evaluation criteria in solid tumors, iCPD = confirmed progressive disease, iUPD = unconfirmed progressive disease, PD = progressive disease, PR = partial remission, RECIST = response evaluation criteria in solid tumors, SA = software-assisted, SD = stable disease, SLD = sum of longest diameters.
