# Supplementary material for: Tumor Response Evaluation Using iRECIST: Feasibility and Reliability of Manual Versus Software-Assisted Assessments
Source: Cancers (Basel). 2024 Feb 29;16(5):993. doi: 10.3390/cancers16050993 (PMC10931003; doi:10.3390/cancers16050993)
Supplement: Supplementary file 1 [file cancers-16-00993-s001.zip › cancers-2882825-supplementary.pdf]

cancers MDPI

Supplementary Material

Figure S1. Tumor response assessment according to iRECIST criteria.

PI Name:	Subject # / Initial:		CT/MRI request date:	Exam date :	RECIST Assessment Date :	Radiologist Initial:
	Study protocol:		CT/ MRI: brain, neck ☐	CT/ MRI: thorax ☐	CT/ MRI: abdomen ☐	CT/ MRI: pelvis ☐
Site Codes:						
1 = Bone	3 = Lung	5 = Abdomen	7 = CNS	9 = Skin/Soft tissue		
2 = Liver	4 = Lymph nodes	6 = Mediastinum	8 = Pelvis	10 = Other		

Localization	Site code			Baseline	FU1	FU2	FU3	FU4	FU5	FU6
Description of target lesions:				SLD (mm)	SLD (mm)	SLD (mm)	SLD (mm)	SLD (mm)	SLD (mm)	SLD (mm)
1										
2										
3										
4										
5										
Sum Assessment				0	0	0	0	0	0	0
Target lesion response (CR, iuPD, icPD, PD, PR, SD):										
Description of non-target lesions										
Sum Assessment				0	0	0	0	0	0	0
Non-target lesion response (CR, PD,non-CR/non-PD)										
Description of new lesions:										
Overall response (CR, iuPD, icPD, PD, PR, SD):										

Signature: ______________________
